# Isolation, Potential Beneficial Properties, and Assessment of Storage Stability of Direct-Fed Microbial Consortia from Wild-Type Chicken Intestine

**DOI:** 10.1007/s12602-024-10387-0

**Published:** 2024-10-25

**Authors:** Haiku D. J. Gómez-Velázquez, Pamela Peña-Medellín, Carlos O. Guzmán-Hernández, Laura González-Dávalos, Alfredo Varela-Echavarría, Armando Shimada, Ofelia Mora

**Affiliations:** 1 Laboratorio de Rumiología y Metabolismo Nutricional (RuMeN), Facultad de Estudios Superiores-Cuautitlán (FESC), Universidad Nacional Autónoma de México (UNAM), 76231 Querétaro, Qro Mexico; 2 Posgrado en Ciencias de La Producción y La Salud Animal, UNAM, Mexico City, Mexico; 3 Instituto de Neurobiología, UNAM, 76231 Querétaro, Qro Mexico

**Keywords:** Microbiota, Probiotics, *Lactobacillus*, Poultry industry, Storage stability

## Abstract

Direct-fed microorganisms (DFM) are recognized as an alternative to antibiotic-based growth promoters in poultry production due to their health benefits. DFM, however, should undergo rigorous safety testing to ensure they meet the criteria to be “Generally Recognized as Safe.” This study assessed eight bacterial consortia (BC) isolated from the ileal and cecal intestinal regions of wild-type chickens, subjecting them to probiotic tests. Subsequently, they were spray- and freeze-dried to evaluate their storage stability for 30 days. BC5-I and BC7-I, isolated from the ileum, emerged as promising DFM, displaying a high content of Lactobacillales using a selective medium and higher susceptibility to Gram-positive and Gram-negative antibiotics. These BC showed a high tolerance to temperature (> 90%), pH > 4 (88–98%), and antagonist effects against *Escherichia coli* and *Salmonella*. BC5-I exhibited superior survival in the simulated gastric conditions and satisfactory intestine mucus adhesion. Freeze-drying was the best method to obtain BC5-I and BC7-I powders, with a survival efficiency of 80.3% and 73.2%, respectively, compared to the beginning of storage. BC5-I presented the lowest cell death rate and prolonged half-life through survival storage kinetics. BC5-I only contained *Lactobacillus*, and *Limosilactobacillus reuteri* was the predominant species in liquid (78.3%) and freeze-dried (59.8%) forms. BC5-I stands out as a promising *Lactobacillus*-based DFM that could improve chicken intestinal health and enhance meat and egg production.

**Supplementary Information:**

The online version contains supplementary material available at 10.1007/s12602-024-10387-0.

## Introduction

Mexico is one of the largest producers of poultry in the world, being fourth and sixth in egg and chicken meat production, respectively [1]. In commercial poultry production, antibiotic growth promoters are the most widely used additives to improve feed conversion, growth performance, and animal health, increasing productivity and profitability [2]. Nevertheless, antimicrobial resistance (AMR) is among the greatest threats to global health, food security, and development.

The World Health Organization and many nations are designing strategies against AMR, reducing or banning the use of antibiotics as feed supplements in livestock production [3]. In this context, plant-based additives, organic acids, prebiotics, and probiotics have been tested as alternative solutions to fight or prevent AMR [2, 4, 5]. The probiotics are highlighted because they also offer significant health benefits and promote animal performance.

Probiotics are “live microorganisms which, when administered in adequate amounts, confer a health benefit to the host” [6]. Probiotics, also known as direct-fed microorganisms (DFM), are known to provide overall protection in animal production. Benefits include modulation of gut microbiota against pathogenic strains, prevention of gastroenteritis, reduction of inflammation and diarrhea, strengthening of the immunological system, and growth promotion [3, 7–9]. DFM and probiotics are interchangeable terms referring to beneficial strains in animal science, yet their applications differ slightly. While probiotics exert a more comprehensive range of advantages as functional foods, the primary function of DFM is to prevent gut infection through the normalization of microbiota thus promoting animal performance [8].

The gut microbiota from the chicken gastrointestinal tract (GIT) has emerged as a potential source of DFM [7, 8, 10]. The two main selection criteria to develop DFM with bacterial consortia (BC) or strains are that they should be safe and produce desirable benefits [9, 11]. It is also advisable to look for potential DFM strains from the intestines of the target host. In addition, the BC or DFM candidates must undergo rigorous testing to achieve inclusion in the Generally Recognized as Safe (GRAS) category. This includes the identification of bacterial strains and assessment of antibiotic resistance, tolerance to various pHs and temperatures, survivability under gastric digestibility conditions, and adhesion to mucus or endothelial cells, as well as their ability to withstand the process of production, manufacturing, distribution, and storage before reaching the target animal [9, 12–14].

The major DFM used in poultry production isolated from the chicken GIT belong to the genera *Lactobacillus*, *Bifidobacterium*, and *Enterococcus* [11, 15]. Some studies have isolated and characterized *Lactobacillus* strains from the caeca and distal ileum of broiler chickens, demonstrating variation of the microbial populations in these regions [15]. Ma et al. [11] evaluated the benefits of supplementation with the strain YPG14 of *L. reuteri* isolated from broiler chicken gut in chicks infected with *Salmonella pullorum*. They demonstrated significant improvement in health status, reduced body weight loss, and increased survival rate of these chicks.

*S. pullorum* causes pullorum disease, which is bird-specific and primarily affects chicks. This disease leads to high morbidity and mortality in young birds, with typical symptoms including anorexia, diarrhea, dehydration, and weakness*.* Consequently, several studies have isolated beneficial DFM strains free from *Salmonella* employing selective media such as Man, Rogosa, and Sharpe (MRS) [13, 16]. The MRS is selective for lactic acid bacteria, including *Lactobacillus*, *Lactococcus*, and *Streptococcus*. In contrast, pathogenic enterobacteria strains such as *Salmonella* and *Escherichia coli* do not grow in this medium. Nevertheless, despite these studies contributing to the knowledge of potential chicken DFM strains, evaluating their viability after storage remains a necessity.

DFM viability must be maintained during long-term storage for probiotic and pharmabiotic applications [13, 17, 18]. Factors such as temperature, oxygen humidity, and dosage form affect the viability of probiotics and, thus, their health-promoting effect [17, 19].

Drying technologies, such as spray-drying, freeze-drying, and cryo-preservation, have been applied to enhance the long-term stability of probiotics [18]. Spray-drying is rapid, continuous, and cost-effective, and its main advantage is that the characteristics of the resulting powder can be easily controlled [17]. On the other hand, freeze-drying is the most suitable and widely used process for removing water for long-term storage of probiotics, although it is expensive [17, 20].

Moreover, adding carriers to the bacterial suspension, such as carbohydrates, proteins, and emulsifiers, is one of the most applied protection strategies to improve drying techniques [17, 18, 20].

Studies on spray- or freeze-drying of DFM isolated from chicken or other species have yielded methods that prolong their storage stability and protect them from adverse environmental and GIT conditions [19, 21–23]. These studies, however, have primarily focused on optimizing the drying method rather than characterizing their potential as DFM [16, 21, 22].

In this work, we isolated a DFM candidate from the ileum and cecum of chickens. Lactic acid bacterial strains were selected through serial cultivation in MRS and characterized by 16 s rRNA sequencing. Additionally, we assessed the effects of the two drying methods mentioned above on the shelf-life and composition of BC. Tests were also conducted to evaluate susceptibility to antibiotics, tolerance to temperature and pH, antagonist effects, mucus adhesion, and simulated gastric conditions. Furthermore, we assessed the storage stability of BC in liquid form and powder obtained by spray- or freeze-drying. This study represents a significant step toward identifying DFM candidates for subsequent in vivo experiments.

## Materials and Methods

### Isolation of Bacterial Consortia and Culture Conditions

Samples were obtained from the intestines of eight clinically healthy wild-type 1-year-old chickens (*Gallus gallus*). The birds were free-range bred (donated from the hatchery of Sergio Estrada, Acequia Blanca, Queretaro, Mexico). This work was approved by the Internal Committee for the Care and Use of Experimental Animals (SICUAE.MC-2021/4–4, UNAM). Chickens were sacrificed humanely by cervical dislocation, and the ileum and cecum were recovered through a scalpel incision. Immediately, the contents of each intestinal sample were stored in 1.5-mL Eppendorf tubes and frozen at − 70 °C. Each sample was cultured in nutritive medium A (NMA) and MRS broth at 37 °C for 24 h under anaerobic conditions until reaching an optical density at 600 nm (OD_600_) of 0.5–0.6, which was measured every 2 h with an HP/Agilent 8453 UV–VIS spectrophotometer (SpectraLab Scientific, USA) to isolate raw bacterial consortia (BC) [24]. Based on our laboratory experience, these growth conditions typically correspond to bacterial concentration of approximately 10^6^ to 10^7^ CFU/mL when the OD falls within the 0.5–0.6 range.

One milliliter of each 24 h culture was mixed with 1 mL of 70% glycerol and stored at − 70 °C until culture for further analysis to evaluate antimicrobial resistance (AMR) genes and for DNA extraction. The microbial population of these eight raw BC growing in an MRS medium (four for each intestinal region) was initially analyzed through 16S rRNA sequencing. Then, based on colony morphology, specific BC was selected to identify lactic acid bacteria by serial cultivation in MRS.

#### Extraction of Genomic and Plasmid DNA from Bacterial Consortia

Aliquots of each raw BC from the ileum and cecum were grown in both, NMA and MRS. Bacterial cells were then concentrated by centrifugation (4000 × g, 10 min), and the pellet was used for the genomic and plasmid DNA extraction. Genomic DNA was extracted using the RBB + C method [25]. Samples (1 mL) were homogenized with zirconium beads and lysis buffer, followed by centrifugation, recovering the supernatant. Next, 260 µL of 10 mM ammonium acetate was added, ice incubated for 5 min, and centrifugated (4 °C, 10 min, 160,000 g). Then, one volume of 98% isopropanol was added to the supernatant, and ice was incubated for 30 min for nucleic acid precipitation. Finally, the isopropanol was recovered, and the pellet was washed with 1 mL of 70% ethanol, and the pellet was dried using a vacuum. Finally, the columns of the QIAamp DNA Mini Kit (QIAGEN, Hilden, Germany) were used for protein removal and DNA purification.

The Wizard Plus SV Miniprep DNA purification kit (Promega, Madison, WI, USA) was used to extract plasmid DNA from all BC samples. Genomic and plasmid DNA concentrations were measured with a Qubit 3.0 fluorometer (Life Technologies, Carlsbad, CA, USA), and DNA integrity was verified by agarose gel electrophoresis.

#### Sequencing of 16 s rRNA of Microbiota

Genomic DNA of the microbiota was used for 16 s rRNA sequencing (RTL Genomics, Lubbock, TX) with the Illumina MiSeq platform. Amplicon sequencing was performed for the V3–V4 region, obtaining 10,000 reads per sample. Data quality control and analyses were performed using the DADA2 model in RStudio [26]. The FASTQ forward and reverse files were merged into a single FASTQ file per sample. Quality control and processing included removing adapters, cutting the sequence reads to 400 bp, and eliminating those smaller than 200 bp. The filtered reads were then aligned with the SILVA database (Silva_nr99_V138.1) to define the amplicon sequence variant (ASV) for taxonomy assignment. In addition, the ASV table was constructed with the DNA sequence reads as an end-product of DADA2. R packages were used to analyze microbiome data. The results of taxonomic composition are expressed as relative abundance (%) at phylum and order level.

### Probiotic Tests

#### Susceptibility to Antibiotics

An antibiogram for Gram( +) and Gram( −) bacteria was performed using a commercial multidisc kit (Multibac ID, Investigación Diagnóstica, CDMX, MX) to evaluate the susceptibility to antibiotics of the eight BC samples. Gram( +)-specific antibiotics were as follows: DC, dicloxacillin (1 µg); CLM, clindamycin (30 µg); E, erythromycin (15 µg); PE, penicillin (10 U); VA, vancomycin (30 µg); TE, tetracycline (30 µg). Gram( −) and ( +)-specific antibiotics were as follows: AM, ampicillin (30 and 10 µg, respectively); CF, cephalothin (30 µg); CFX, cefotaxime (30 µg); CPF, ciprofloxacin (5 µg); GE, gentamicin (10 µg); SXT, sulfamethoxazole/trimethoprim (25 µg). Each BC was grown in 15 mL of MRS medium to OD_600_ 0.5–0.6, and then 100 µL of each culture was seeded onto Mueller-Hinton agar plates. Antibiotic multidiscs were placed on these plates, and they were incubated anaerobically at 37 °C for 24 h. After incubation, the halo of the inhibition was measured. The results are expressed as resistant (R), intermediate (I), and susceptible (S) according to the manufacturer’s instructions.

#### Genomic and Plasmid AMR Genes

Since BC5 and BC7 from the ileum (BC5-I & BC7-I) displayed lower susceptibility to antibiotics, they were selected for further analysis. Likewise, the NMA broth was not used in subsequent studies since BC did not grow satisfactorily. To assess the presence of antibiotic resistance genes in BC5-I and BC7-I isolates, genomic DNA and plasmid DNA were extracted as described above, and PCR was performed for Tet R, Tet-M, Tet-W, AM, CL, E, Aminoglycosides (AMG), Van A, and Van B with the primers and conditions indicated in Table S1.

#### Isolation and Selection of Lactic Acid *Bacteria* as DMF Candidates

To ensure the absence of *Salmonella*, enterobacteria, or other pathogen strains in the DFM, BC5-I and BC7-I, which had the highest sensitivity to antibiotics, were cultivated serially in an MRS medium to isolate lactic acid bacteria based on colony morphology and environmental scanning electron microscopy (data not shown). Subsequently, these BC were subjected to further evaluation for probiotic tests.

#### Temperature Tolerance Test

BC5-I and BC7-I isolates were cultivated on MRS broth as indicated in the “Isolation of Bacterial Consortia and Culture Conditions” section, and 1 mL aliquots (OD_600_ = 0.5–0.6) were inoculated in 50 mL of MRS broth at different temperatures (30, 37, and 45 °C) to assess their temperature tolerance [13]. Serial dilutions of each culture were then seeded on MRS agar plates and cultured in anaerobiosis at 37 °C for 24 h to quantify viable bacteria. The results are expressed as Log_10_ CFU/mL.

#### Resistance to pH

BC5-I and BC7-I isolates were cultivated in MRS broth as indicated in the “Isolation of Bacterial Consortia and Culture Conditions” section, and 1 mL aliquots (OD_600_ = 0.5–0.6) were inoculated in 50 mL of MRS broth in triplicate with different pH values (2, 3, 4, 5, 5.5, 6, 6.5, and 7) to evaluate pH resistance. The broth at pH 6.5 was used as a control. The quantitative viable bacteria were determined as previously described [9, 24]. The results are expressed as the survival percentage (%) of colonies-formed unit per mL (CFU/mL) at each pH in comparison to control.

#### Resistant to Bile Salt

The assessment of bile salt resistance was done with 100 µL of BC culture (OD_600_ nm = 0.5–0.6) inoculated in MRS and NMA broths at pH 7.5 with or without 0.3% ox bile salt and grown anaerobically at 37 °C. The growth curve of each BC culture was monitored by measuring OD_600_ nm every 2 h up to 6 h. The cultures were then diluted by tenfold serial dilutions to 1:1 × 10^4^ and 1:1 × 10^5^ (v/v, ratio), plated on MRS and NMA agar, and incubated anaerobically at 37 °C for 24 h. The survival rate was expressed as a percentage of CFU/mL [24].

#### Antagonistic Activity

Two pathogenic strains (*Salmonella typhimurium* ATCC 19028 and *E. coli* ATCC 11229, donated by the Facultad de Medicina, Universidad Autónoma de Quéretaro, Querétaro, MX) were used to evaluate the bacterial antagonism of BC5-I and BC7-I cultures by the disc diffusion method [27]. Pathogenic strains were grown in LB broth, and 100 µL was seeded on Mueller-Hinton agar plates to create a uniform lawn. BC was grown in MRS broth as indicated in the “Isolation of Bacterial Consortia and Culture Conditions” section, 10 mL of culture was concentrated 50-fold by centrifugation (1000 × g, 5 min), and the concentrated cell suspension was used to saturate sterile paper discs, which were then placed on the lawns of each of the pathogenic strains. After incubation at 37 °C for 24 h, the growth inhibition zone (mm) was measured. The antagonistic activity was considered positive when the average of halo inhibition was ≥ 10 mm.

#### Simulated Gastric Digestion

BC5-I and BC7-I isolates were grown in MRS broth as indicated in the “Isolation of Bacterial Consortia and Culture Conditions” section, concentrated, and used for the simulated gastric digestion (SGD) assay [11, 13]. The BC samples were added to 10 mL of artificial gastric fluid (3.3 g/L pepsin, 10 mM PBS, pH 2.5) and were incubated for 120 min at 37 °C. The viable bacteria were counted on MRS agar plates at intervals of 30 min. The SGD percentage resistance was reported as Log_10_ CFU/mL and expressed as resistant (> 67%), tolerant (34–66%), and sensible (< 33%) according to the following equation:

% SGD = [Initial bacteria (Log_10_ CFU/mL)/final bacteria (Log_10_ CFU/mL)] × 100.

#### Adhesion to Mucin Assay

Chicken small intestine mucin was obtained according to Vélez et al. [28] and used to evaluate the adhesion of BC5-I isolate owing to its SGD resistance. Briefly, the mucosal bacteria were labeled by incubation in 250 µL of 5-([4,6-dichlorotriazin-2-yl] amino) fluorescein hydrochloride (5-DTAF, Sigma-Aldrich, USA) in 500 µL of PBS at 60 °C for 2 h and washed three times by resuspension in PBS and collected by centrifugation at 10,000 × g 10 min at 4 °C. At the same time, BC5-I cells (100 µL, OD_600_ 0.5–0.6) were labeled by incubation in 4′,6-diamidino-2-phenylindole dihydrochloride, 2-(4-amidinophenyl)-6-indolecarbamidine dihydrochloride (DAPI, Sigma-Aldrich, St Louis, MO, USA) in PBS (diluted 1:1000 and 1:100,000) at 37 °C for 2 h [24]. Then, 10 µL of labeled BC cells was mixed with 200 mL of labeled chicken mucins, incubated at 37 °C for 2 h, washed by centrifugation and resuspension in PBS three times, diluted 1:100,000 in PBS, and fixed with paraformaldehyde (4%) on microscope slides. For microscopic analysis, the MosaiX module for the ApoTome system with the 40 × /1.30 DIC (UV) VIS-IR M27 Plan-Apochromat oil immersion objective was used to obtain a full mosaic image (1 mm^2^). The MosaiX system (Carl Zeiss, Jena, Germany) collected and assembled three individual image stacks for each histological slide. Viable bacteria that adhered to the mucins were observed under a microscope and analyzed using ImageJ64 and FIJI software [29] to quantify viable bacteria on the 2D images. Particles were quantified using Yen’s image thresholding method [30].

### Preservation of BC Through Drying Methods

BC5-I and BC7-I isolates were cultured in MRS broth as described in the “Isolation of Bacterial Consortia and Culture Conditions” section and then freeze- or spray-dried, followed by an evaluation of their viability.

#### Freeze-Drying

Three 50 mL aliquots of 24 h cultures of BC5-I and BC7-I were concentrated by centrifugation (4000 × g, 10 min). The pellets were diluted with 75 mL of 3% sterile skim milk in aseptic conditions. Next, each mixture was snap-frozen in a bath of powdered CO_2_ and acetone. Mixtures were then lyophilized (Freeze Dryer 5, Labconco, Fisher Sci, Hampton, NH, USA) for 24 h to obtain BC powders [18, 20]. These BC powders were stored and protected from light at 4 °C until their analysis.

#### Spray-Drying

One liter of BC culture was mixed with 80% whey protein concentrate (WPC80) and Arabic gum (AG) solution as carrier agents. Previously, 187.5 g of WPC80 was dissolved in 250 mL of distilled water with magnetic stirring adjusting pH to 3.5, which were mixed with AG solution (500 g/125 mL) for 2 h. The spray-drying process was performed using a laboratory-scale spray drier (Büchi, B-290, Flawil, CH) with a 1 L/h drying rate. The mixture was fed into the drying chamber at room temperature (25 °C) with a peristaltic pump with a flow rate of 2.3 mL/min. The inlet air temperature was 180 and 80 °C, and the outlet air temperature ranged from 46 to 50 °C. A drying air flow rate of 0.36 m^3^/h and an aspirator flow rate of 95% (~ 33 m^3^/h) were used. The conditions were established based on preliminary experiments developed in the Laboratorio de Biotecnología Acuícola y Acuicultura of the Universidad Michoacana de San Nicolas Hidalgo (UMSH, LANMDA-CONACYT). The spray-dried BC powders were homogenized and stored at 4 °C until viability and storage analysis.

#### Identification of *Lactobacillus* Strains and 16 s rRNA Sequencing of BC Powders

Genomic DNA was extracted from dried BC powders (200 mg) as described in the “Extraction of Genomic and Plasmid DNA from Bacterial Consortia” section. A PCR reaction was performed on the BC5-I and BC7-I genomic DNA to confirm the presence of specific bacteria at the species level associated with probiotic activities. The primers were designed for *Lactobacillus acidophilus*, *Lacticaseibacillus casei* (*L. casei*), *L. helveticus*, *Lacticaseibacillus rhamnosus* (*L. rhamnosus*), and *L. cremoris*. Primer sequences and PCR conditions are shown in Table S2.

In addition, 16S rRNA sequencing was performed for both liquid and dried forms of BC, and analysis was carried out as described above.

#### The Relative Proportion of *Lactobacillus* in BC Powders by Quantitative PCR

Total bacteria, *Lactobacillus*, and enterobacteria proportions were determined by quantitative PCR (qPCR) in BC5-I powders as they had the highest storage stability. Primer sequence and qPCR conditions are shown in Table S3. A total of 50 ng of genomic DNA measured in a NanoDrop 1000 spectrophotometer was used for the qPCR reaction [31]. The primer products were quantified using a StepOne™ Real-Time PCR System (Applied Biosystems, Thermo Fisher, Sci, Waltham, MA, USA), and the relative abundance of the DNA target was calculated according to Bacchetti De Gregorio et al. [31].

### Viability and Storage Stability of BC Powders Assay

BC powders were resuspended in 1 mL of distilled water, and the viable bacteria were determined as described above. The survival percentage efficiency ratios of BC powders obtained from spray- or freeze-drying were calculated from the viable bacteria count after and before drying (Log CFU/g powder).

For survivor stability, BC5-I and BC7-I cells in liquid and powder forms were stored in Falcon tubes covered with aluminum foil for 30 days at 4 °C. Viable bacteria counts were determined at timed intervals of 0, 7, 14, 21, and 30 days. The results are expressed as Log_10_ CFU/mL or Log_10_ CFU/g powder.

#### Kinetic Parameters

The bacteria survival and the storage stability data were subjected to a first-order kinetic model. The kinetic rate constant (*k*) of each sample by the effect of storage conditions was calculated using the following expression:

*N*_0_ = *N*_t_ *exp(− *kt*).

where *t* is the storage time (days), *k* is the first-order rate constant (days^−1^), and *N*_0_ and *N*_t_ are the CFU/mL (or CFU/g) at the initial time of the study and in the function of the storage time, respectively [32]. The half-life was calculated using the following expression:

*t*_1/2_ = Ln (2)/*k.*

where *k* is the respective kinetic constant.

### Statistical Analysis

Each experiment was independently run in triplicate, and data are expressed as mean ± SEM. Data from BC microbiota 16S rRNA were analyzed and visualized using RStudio software (http://www.rstudio.com) with the packages of the phyloseq library [33] and Bioconductor. Differences between results were determined by one-way analysis of variance and comparative mean differences by the Tukey test at *p* < 0.05 using Minitab Statistical Software (version 18). The kinetics data of storage stability were subjected to linear or nonlinear regression analysis to fit them to one-phase decay curves using statistical tools in GraphPad Prism software version 6 (San Diego, CA, USA). Their validation goodness of fit was by the determinate coefficients (*R*^2^). In addition, some graphics were produced with RStudio and GraphPad Prism software.

## Results

### Characterization of Chicken Intestine Bacterial Consortia

**Fig. 1:**
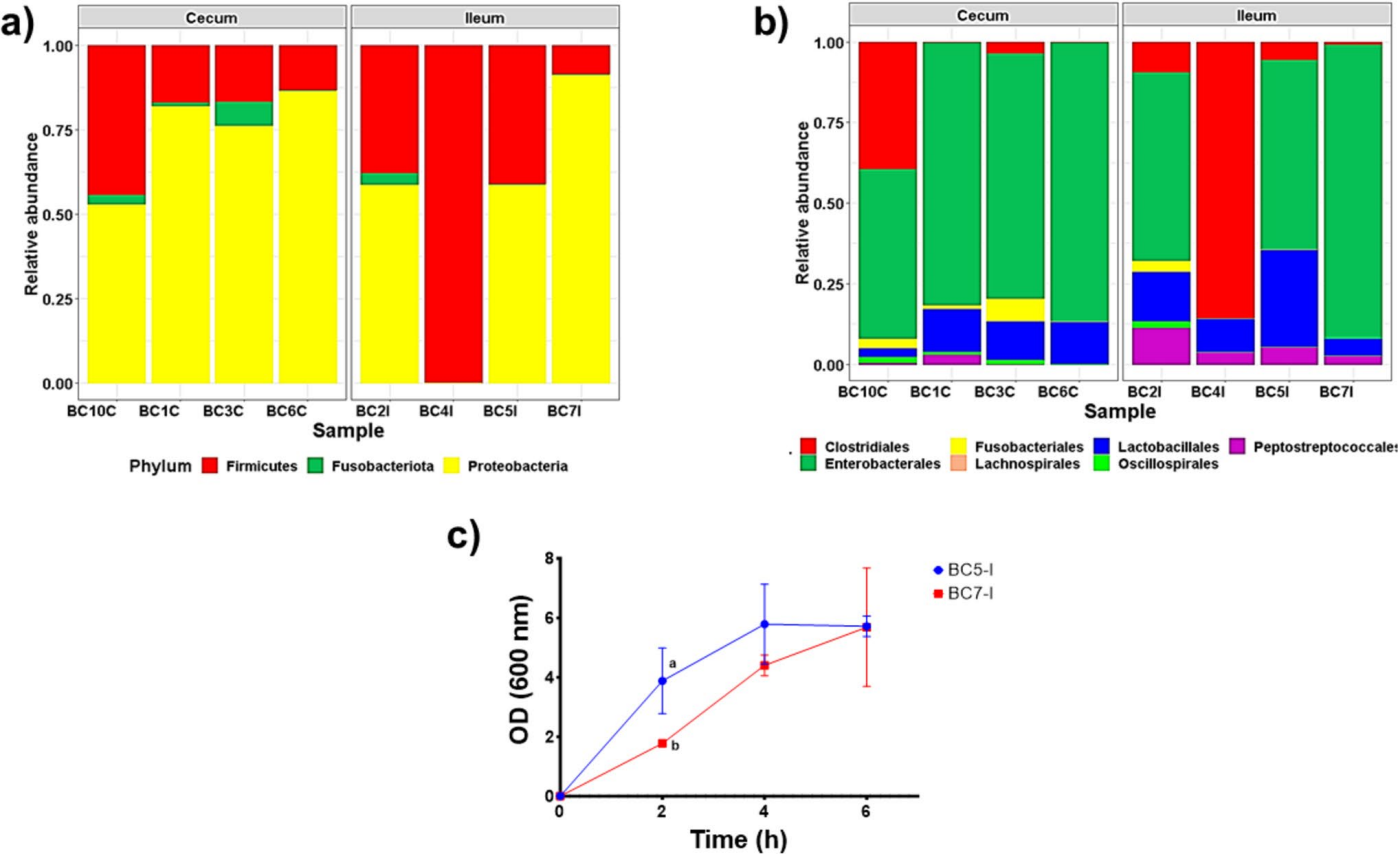
Relative abundance at phylum (**a**) and order (**b**) levels, as well as the growth rate in exponential phase (**c**) of bacterial consortia isolation from ileum and cecum of chicken intestine cultivated in MRS broth. BC, bacterial consortium; I, ileum; C, cecum; OD, optical density at 600 nm. The results are expressed as means values and SEM (*n* = 3). Different letters at the same time indicate significant differences by the Tukey test (*p* < 0.05)

In this work, we isolated intestinal bacterial consortia from chicken ileum and cecum, which were analyzed by sequencing of 16S rRNA (Fig. 1). Three predominant phyla were detected in the isolates from both regions: Proteobacteria showed the highest relative abundance in samples from the ileum (58–90%) and cecum (52–86%), followed by Firmicutes (10–41% and 13–44%, respectively), and Fusobacteriota (0–7%) (Fig. 1a). At the order level, ileum and cecum samples revealed the presence of Enterobacterales as the major group (52–86% and 58–91%, respectively), followed by Clostridiales (10–85% and 10–39%, respectively), and Lactobacillales (10–30% and 2–15%, respectively) (Fig. 1b). Firmicutes are related to beneficial bacteria, including the *Lactobacillus* which are used as the most common probiotic. In this sense, BC5 isolated from the ileum (BC5-I) presented the highest relative abundance of Lactobacillales (30%).

### Probiotic Tests

#### Susceptibility to Antibiotics

Raw bacterial consortia isolated from chicken ileum and cecum grew in NMA and MRS media. Growth of BC from both intestinal regions was better in MRS (Table 1). In contrast, most BC samples failed to grow in NMA. Therefore, subsequent determinations were done only on MRS cultures.

**Table 1:** Antibiograms susceptibility profiles to Gram( −) and Gram( +) antibiotics of potential probiotic bacteria consortia (BC) isolated from chicken ileum and cecum intestine grown on MRS and NMA broths

Broth	Nutritive medium A (NMA)							Man, Rogosa, and Sharpe (MRS)						
Samples	Cecum				Ileum			Cecum				Ileum		
	BC10-C	BC1-C	BC3-C	BC6-C	BC2-I	BC 5-I	BC-7-I	BC10-C	BC1-C	BC3-C	BC6-C	BC2-I	BC 5-I	BC-7-I
**Antibiotics Gram( −)**														
** AK**	NG	R	NG	R	R	R	NG	R	R	R	R	R	R	R
** AM**	NG	R	NG	R	R	S	NG	R	R	R	R	R	S	S
** CB**	NG	R	NG	R	R	I	NG	R	R	R	R	R	R	R
** CF**	NG	R	NG	R	R	R	NG	R	R	R	R	R	R	R
** CFX**	NG	R	NG	R	R	R	NG	R	R	R	R	R	R	R
** CPF**	NG	R	NG	R	R	R	NG	R	R	R	R	R	R	I
** CL**	NG	R	NG	R	R	R	NG	R	R	R	R	R	S	S
** GE**	NG	R	NG	R	R	R	NG	R	R	R	R	R	R	R
** NET**	NG	R	NG	R	R	R	NG	R	R	R	R	R	R	R
** NF**	NG	S	NG	R	S	R	NG	R	I	S	I	R	S	S
** NOF**	NG	R	NG	R	R	R	NG	R	R	R	R	R	S	S
** STX**	NG	R	NG	R	R	R	NG	R	R	R	R	R	R	S
**Antibiotics Gram( +)**														
** DC**	NG	R	R	NG	R	R	R	R	R	R	R	R	R	R
** AM**	NG	R	R	NG	R	I	I	R	R	R	R	R	I	I
** CLM**	NG	R	R	NG	R	R	R	R	R	R	R	R	R	R
** CF**	NG	R	R	NG	R	R	R	R	R	R	R	R	R	R
** CFX**	NG	R	I	NG	S	I	I	R	R	R	I	R	S	I
** CPF**	NG	R	I	NG	R	S	I	R	R	R	R	R	S	I
** E**	NG	R	R	NG	R	R	R	R	R	R	R	R	R	R
** GE**	NG	R	R	NG	R	R	R	R	R	R	R	R	R	R
** PE**	NG	R	R	NG	R	R	R	R	R	R	R	R	R	R
** TE**	NG	R	R	NG	R	R	R	R	R	R	R	R	R	R
** VA**	NG	R	R	NG	R	R	R	R	R	R	R	R	R	R
** SXT**	NG	R	R	NG	R	R	R	R	R	R	R	R	R	R

The results are expressed as resistant (R), intermediate (I), and susceptible (S) according to the manufacturer’s instructions. Gram-negative and Gram-positive specific: AM, ampicillin (30 and 10 µg, respectively); CF, cephalothin (30 µg); CFX, cefotaxime (30 µg); CPF, ciprofloxacin (5 µg); GE, gentamicin (10 µg); SXT, sulfamethoxazole/trimethoprim (25 µg).

*NG* non-growth, *BC-I and -C* bacterial consortia from ileum and cecum, *AK* amikacin (30 µg), *CB* carbenicillin (100 µg), *CL* chloramphenicol (30 µg), *NET* netilmicin (30 µg), *NF* nitrofurantoin (300 µg), *NOF* norfloxacin (10 µg), *DC* dicloxacillin (1 µg), *CLM* clindamycin (30 µg), *E* erythromycin (15 µg), *PE* penicillin (10 U), *VA* vancomycin (30 µg), *TE* tetracycline (30 µg).

Moreover, antibiograms revealed that BC from the ileum were more susceptible to antibiotics than those from the caecum. BC5-I and BC7-I samples, isolated from the ileum, were susceptible to the Gram( +) antibiotics AM, CFX, and CPF and the Gram( −) antibiotics AM, CPF, CL, NF, NOF, and STX. In contrast, BC from caecum only showed susceptibility to nitrofurantoin, a Gram( −) antibiotic. Additionally, almost all BC samples were susceptible to nitrofurantoin (300 µg, NF), resulting in the most effective Gram( −) antibiotic, while cefotaxime (30 µg, CFX) was the most effective Gram( +) antibiotic. Overall, the BC5-I and BC7-I were the most susceptible to antibiotics. Hence, they were selected for the following determinations.

#### BC Growth Rate

BC were selected and isolated as DFM candidates using an MRS medium based on their colony morphology. Their colonies ranged from small to medium and were circular, smooth, and convex with a shiny surface. They exhibited yellow, cream, or whitish coloration. The BC presented *Bacillus*-type morphologies that were corroborated using environment scanning electron microscopy (Gómez-Velazquez et al. in press). The growth rates in the MRS broth of BC5-I showed the highest values at 2 h, in contrast to BC7-I, which reached its peak during the exponential (Fig. 1c). Nevertheless, after 6 h of growth, both BC from ileum samples presented similar values ranging from OD_600_ 4.66 to 5.68 without significant changes. After the 8th hour, the growth rate plateaued, indicating the transition to the stationary phase. Based on these results, BC5-I and BC7-I were selected to continue with the probiotic test since they exhibited the highest antibiotic susceptibility. Additionally, they were used to evaluate the effects of drying methods and subsequent molecular sequencing.

#### Antibiotic Resistance Genes in Genomes or Plasmids

**Fig. 2:**
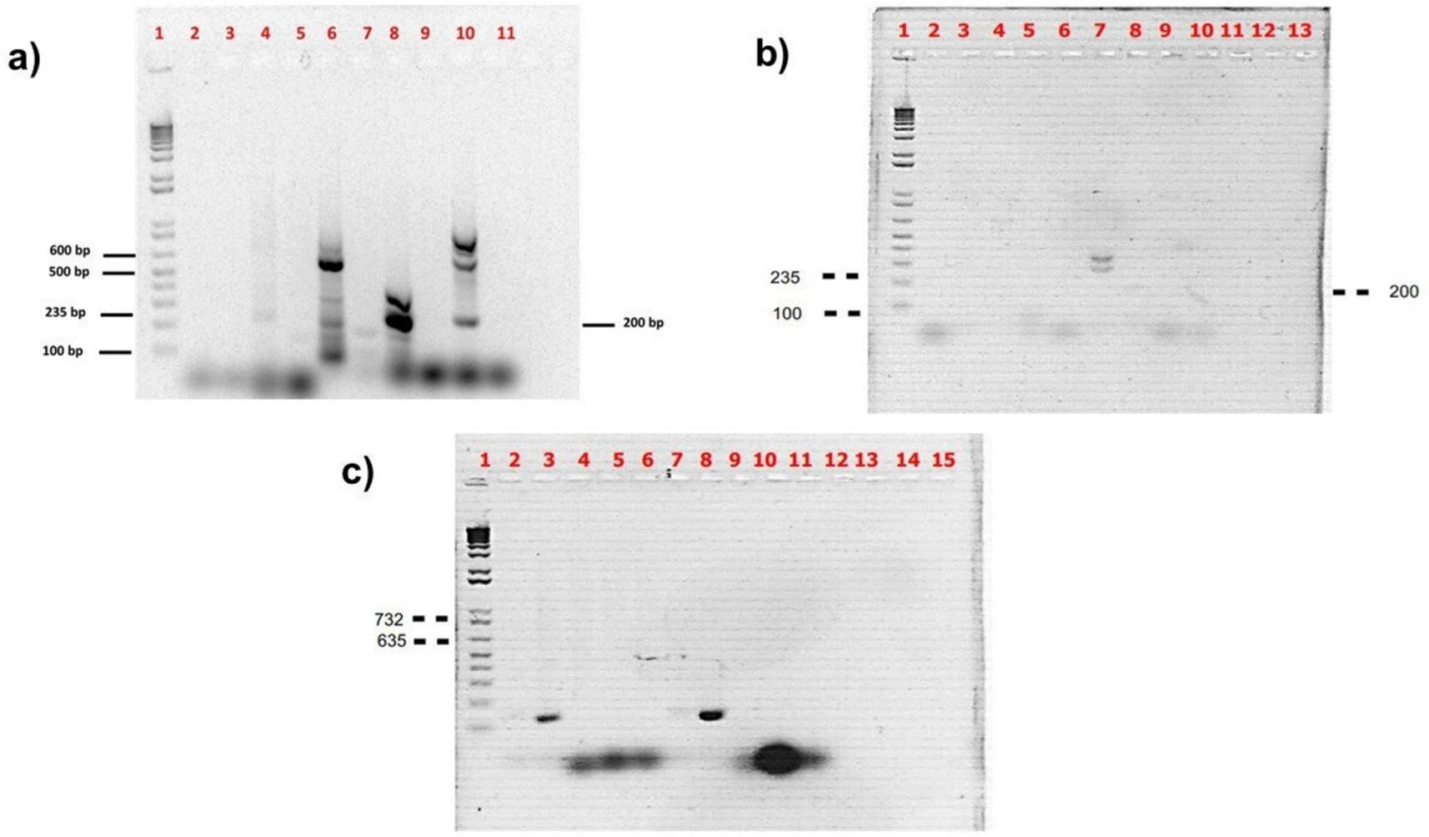
PCR products of plasmid and genomic genes for antibiotic resistance from BC-5 and BC-7 isolated from chicken ileum intestine (I) inoculated in MRS broth. Plasmid gene products of AM, CPF, E, and aminoglycosides (AMG) antibiotic resistance for BC5-I: 1, 1 kb Plus DNA ladder; 2, negative control (without DNA); 3, AM; 4, CPF; 5, E; and 6, AMG, and BC7-I: 7, negative control; 8, AM; 9, CPF; 10, E; and 11, AMG (**a**). Genomic genes for AM, CPF, E, and aminoglycoside (AMG) antibiotic resistance for BC5-I: 1, 1 kb Plus DNA ladder; 2, negative control; 3, AM; 4, CPF; 5, E; and 6, AMG, and BC7-I: 7, AM; 8, negative control; 9, CPF; 10, E for BC5-I; and 11, AMG (**b**). Genomic and plasmid gene products for vancomycin A and B antibiotic resistance for BC5-I: 1, 1 kb Plus DNA ladder; 2, negative control; 3, Van A plasmid; 4, Van B plasmid; 8, Van A genomic; and 9, Van B genomic, and BC7-I: 2, negative control; 5, Van A genomic; 6, Van B genomic; 10, Van A genomic; and 11, Van B genomic (**c**)

The genes of AM, CPF, E, AMG, VA, and VB responsible for AMR were evaluated using PCR at the plasmid and genomic levels in the BC5-I and BC7-I samples (Fig. 2). BC5-I contained resistant genes to AMG and VA at the plasmid level and VB at the genomic level, whereas BC7-I, AM, and AMG were observed in plasmid DNA and VA and AM at the genomic level, respectively.

#### Temperature Tolerance

**Fig. 3:**
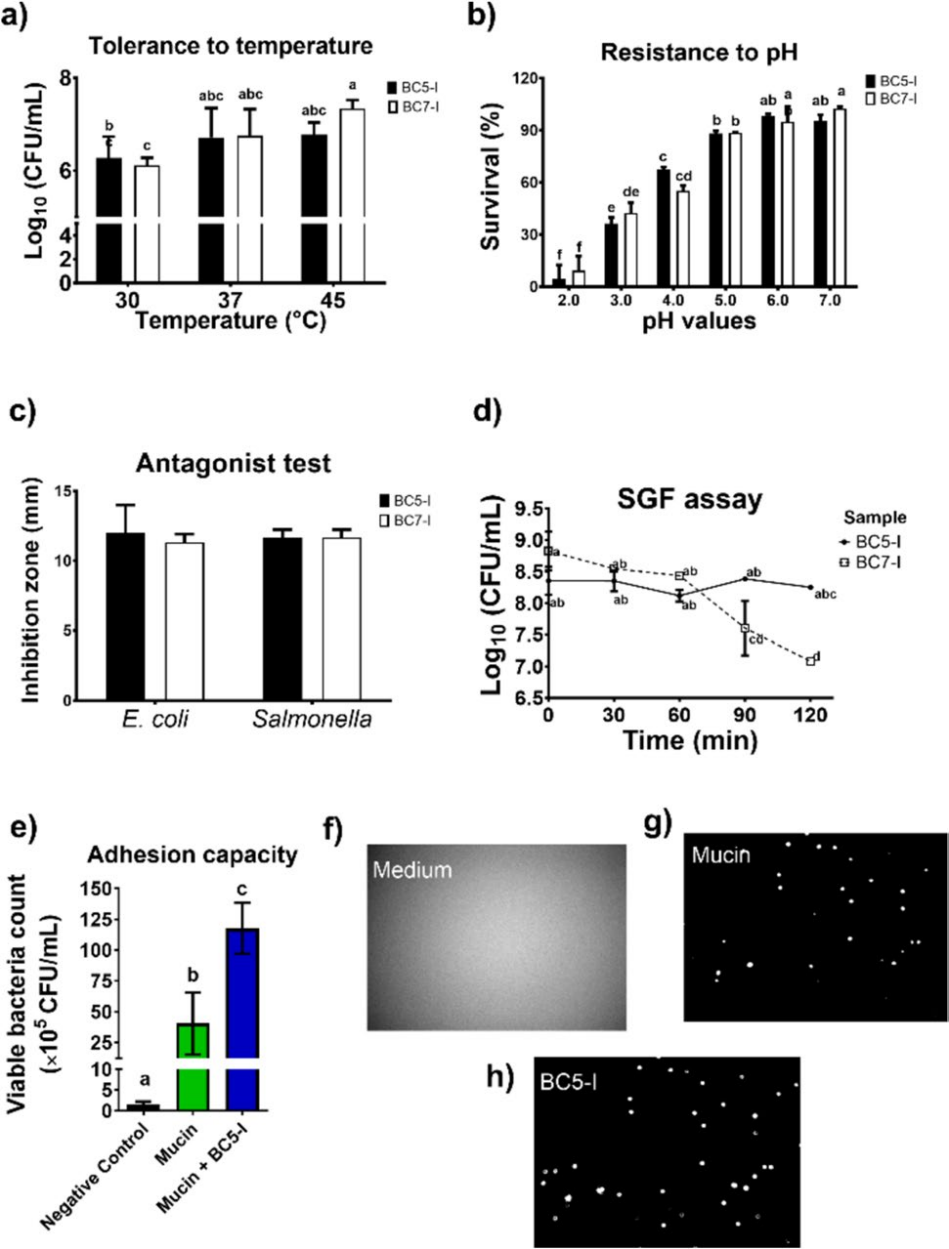
Probiotic test of bacterial consortia (BC) isolated from the chicken ileum intestine (I) and inoculated in MRS broth. Tolerance to temperature tested at 30, 37, and 45 °C (**a**). Survival to different pH values (**b**). Antagonist effects against *E. coli* and *Salmonella* (**c**). Simulated gastric digestion (SGD) assay (**d**). Assay of adhesion capacity to chicken mucin: viable bacterial count (**e**), images of MRS medium (negative control, **f**), mucosal host bacteria (green color, **g**), and BC5-I bound to intestinal mucin (blue color, **h**). The arrows represent BC5-I cells adhered to intestine mucin. The results are expressed as mean values and SEM (*n* = 3). Different letters indicate significant differences among groups by the Tukey test (*p* < 0.05)

BC5-I and BC7-I presented tolerance to the temperature evaluated at 30, 37, and 45 °C (Fig. 3a). Overall, BC5-I had the highest tolerance to temperature, with similar viability values across different temperatures. In contrast, BC7-I was slightly more susceptible to the effects of these temperatures. It had the highest viability values at 45 °C and the lowest at 30 °C (15% less) compared to BC5-I. Nonetheless, in the control temperature of 37 °C, no differences were found between BC samples.

#### Resistance to pH

Confronting probiotics against in vitro pH tolerance tests is crucial to determine whether they can survive and maintain their viability under harsh conditions in the gastrointestinal tract, acidity conditions in the stomach, the transition to neutral pH in the small intestine, and fluctuating conditions in the large intestine.

Both bacterial consortia from the ileum showed significant survival changes depending on pH values, oscillating from 4.5 to 102% compared to those grown in control pH at 6.5 (Fig. 3b). Low pH values affected the growth of both BC5-I and BC7-I, showing the lowest survival at pH 2 (4.5 and 9.4%, respectively), which increased at pH 3 and pH 4, oscillating from 36 to 67% and 42 to 55%, respectively, without significant differences among samples at the same pH. Nevertheless, for pH higher than 5, there was no statistically significant difference between the groups. In addition, the best pH values for BC growth were 6 and 7, with 96 to 102% survival.

#### Resistant to Bile Salt

Both BC5-I and BC7-I samples showed similar tolerance to bile salts of 27 and 25% of survivors, without significant differences, after 6 h in 0.3% bile salt concentrations.

#### Antagonist Activity

Both BC5-I and BC7-I samples showed an inhibition zone larger than 10 mm with *E. coli* and *Salmonella*, revealing positive inhibition without statistically significant values between groups (Fig. 3c).

#### Simulated Gastric Digestibility

BC samples were subjected to in vitro simulated gastric digestibility (SGD) to reproduce some of the conditions of their passage through the intestinal tract (Fig. 3d). After SGD for 2 h, the viable bacteria count of BC7-I significantly decreased from 7.98 × 10^8^ to 1.2 × 10^7^ CFU/mL, revealing a probiotic potential to the gastric condition with a 20% reduction. In contrast, the viable bacteria count of BC5-I was maintained from 2.49 to 1.78 × 10^8^ CFU/mL, with a slight reduction of 1.2%, showing resistance to gastric conditions.

#### Mucin Adhesion Capacity

Adhesion capacity to chicken intestinal mucin was tested for BC5-I, as it had the highest survival in the SGD assay (Fig. 3e, f). The isolate BC5-I presented significant adhesion capacity, colonizing the intestinal mucosa with 2.9-fold more viable bacteria than the initial mucosal host bacteria (Fig. 3e, g, and h).

#### BC Preservation and Storage Stability

Freeze-drying and spray-drying were assessed as preserving methods of BC after evaluating their liquid stability.

#### Liquid BC Storage Stability

**Fig. 4:**
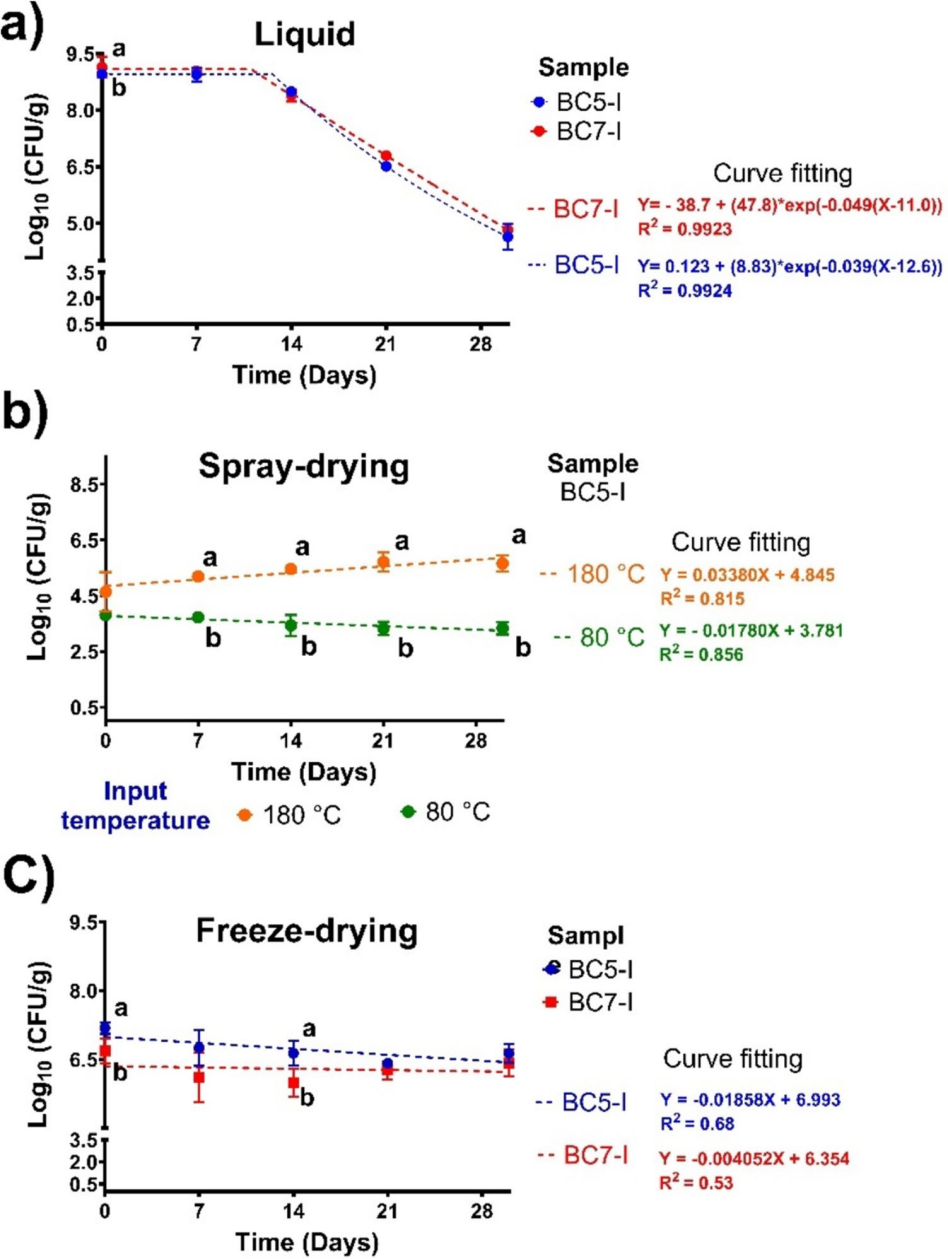
Survival storage stability (4 °C for 30 days) of chicken ileum bacterial consortia (BC5-I and BC7-I) using spray-drying and freeze-drying as conservation methods: Liquid probiotics (**a**); freeze-dried powders using 3% skim milk as a cryoprotectant (**b**) and spray-dried powders (BC5-I) using GA and WP80 as carried agents (1:1 m/v) at two drying input temperatures of 180 and 80 °C (**c**). The results are expressed as mean values and SEM (*n* = 3). Different letters at the same time indicate statistical differences by the Tukey test (*p* < 0.05)

Before producing BC dry powders, the liquid preparations of BC5-I and BC7-I contained 9.2 × 10^8^ and 1.6 × 10^9^ CFU/mL, respectively. As shown in Fig. 4a, the storage stability of both liquid BC experimental data was fitted to a plateau followed by a one-phase decay model (*R*^2^ > 0.99) as a nonlinear regression model. Throughout 14 days of storage, their bacterial viabilities slightly decreased or remained stable (Fig. 4a); after this time, the viabilities of both BC showed a critical decrease down to about 3.6 Log CFU/mL at 30 days of storage (one-phase decay). The liquid BC5-I showed a higher kinetic rate constant (*k* = 0.0165 days^−1^) than BC7-I (*k* = 0.0156 days^−1^), resulting in a lower half-life (43.32 and 44.46 days, respectively).

#### Dried Powders Storage Stability

Both freeze- and spray-drying methods maintained the viability of the BC samples through storage (Fig. 4b, c). In addition, both probiotic dry products presented low moisture content, ranging from 2 to 6%. However, at the beginning of storage, both methods reduced viability compared to that observed in liquid form.

Regarding spray-drying, only the BC5-I powders obtained at input temperatures of 180 and 80 °C had good stability, which was not the case for BC7-I. Thus, only the storage stability of BC5-I powders was evaluated (Fig. 4b). The viable bacteria of these powders, produced at high- and low-input temperatures (7.8 × 10^4^ and 6.6 × 10^3^ CFU/g, respectively) decreased by four and five orders of magnitude, respectively, compared to those from liquid BC5-I before drying (9.2 × 10^8^ CFU/mL). Thus, their survival efficiency percentage (% Log CFU/g) was 51.8 and 41.7% for high- and low-input temperatures, respectively.

In storage, BC5-I powders produced at high input temperature showed a significantly higher viable bacterial count (~ 2 orders of magnitude), which remained constant throughout the storage time compared to those produced at the low-input temperature (Fig. 4b). The viability of the latter decreased slightly by 12% at the end of storage time. These data were fitted to a linear regression curve, resulting in *k* values of 0.034 and 0.018 days^−1^ for the high and low input temperature with an *R*^2^ of 0.815 and 0.856, respectively. The *t*_1/2_ was higher in the BC5-I powders produced at low than at high input temperatures (38.5 and 20.38 days, respectively).

Both BC powders obtained by freeze-drying were found to have good stability. At the initial storage time, the viable bacterial count of BC5-I and BC7-I powders decreased by two and three orders of magnitude compared to those in liquid form. The survival efficiency for C5-I and BC7-I powders was 80.3% and 73.2%, respectively. BC5-I powders showed significantly more viable bacteria (7.18 Log CFU/mL) than BC7-I (6.68 Log CFU/mL).

Regarding the storage time, the viability of BC5-I and BC7-I freeze-dried powders decreased throughout 7 days of storage (8 and 10%, respectively), which remained at 14 days (6.9 and 5.8 Log UFC/g, respectively) and showed significant differences between groups (Fig. 4c). At 21 days, these viabilities decreased by 11% and 9% compared to those at the initial storage time, respectively, without significant differences between BC powders. At the end of storage, the viability of BC5-I and BC7-I was similar to that at 21 days, with 90 and 92% remaining. Similarly, these data were fitted to a linear regression curve, resulting in *k* values of 0.004 and 0.016 days^−1^ for the BC5-I and BC7-I with an *R*^2^ of 0.68 and 0.53, respectively. Concerning the *t*_1/2_, it was longer for BC5-I with values of 173.3 days, while for BC-7I, it was 43.32 days. Therefore, the freeze-drying method provides the best protection for both BC compared to spray-drying at the beginning and across storage in our experimental conditions. Owing to these results, the freeze-dried powders were selected for the following determination.

#### Identification of *Lactobacillus* at the Species Level by PCR

**Fig. 5:**
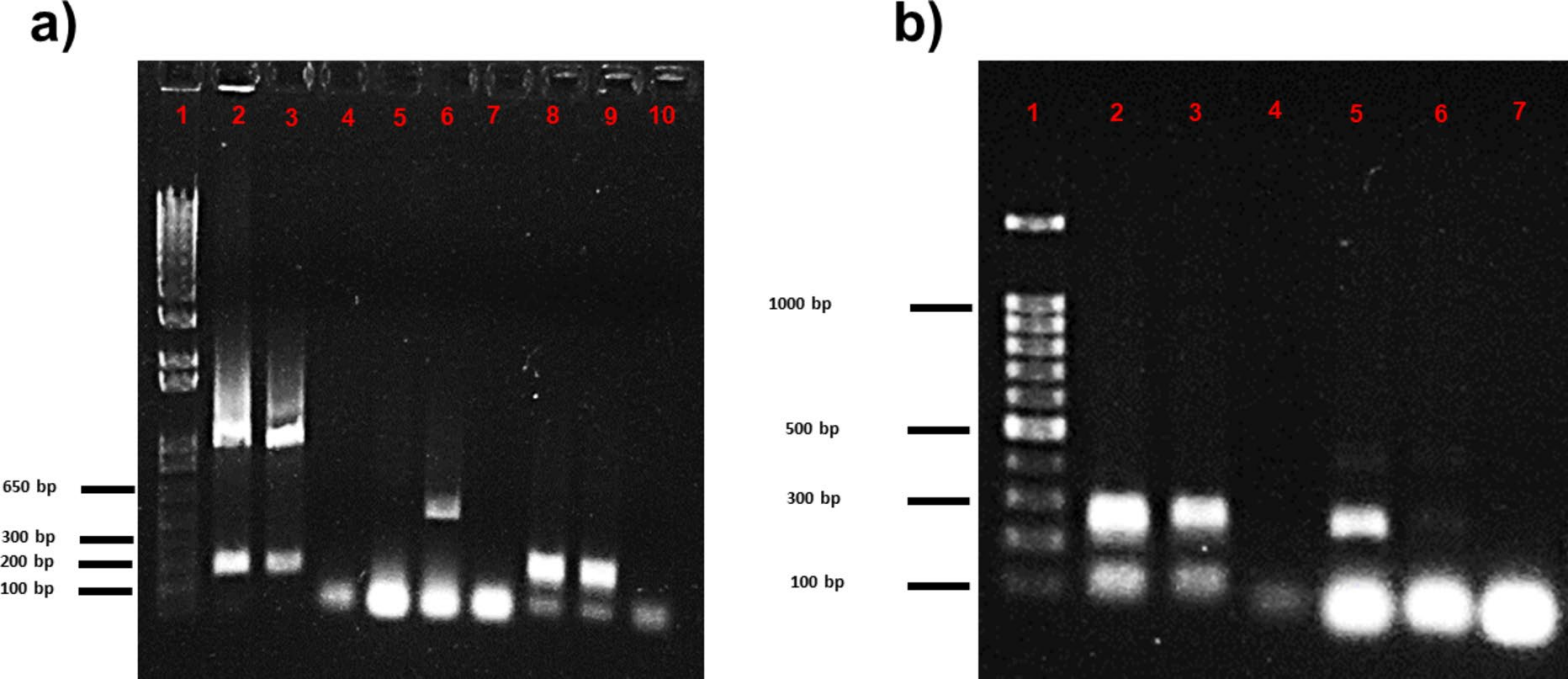
Specific *Lactobacillus* PCR products of genomic DNA from freeze-dried BC5-I and BC7-I powders. PCR products for *L. acidophilus* (1, 1 kb Plus DNA ladder; 2, BC5-I; 3, BC7-I; and 4, negative control), *L. rhamnosus* (5, BC5-I; 6, BC7-I; and 7, negative control), and *L. casei* (8, *BC5-I*; 9, BC7-I; and 10, negative control) (**a**); PCR products for *L. helveticus* (1, 100 bp Plus DNA ladder; 2, BC5-I; 3, BC7-I; and 4, negative control) and *L. cremoris* (5, BC5-I; 6, BC7-I; and 7, negative control) (**b**)

**Fig.6:**
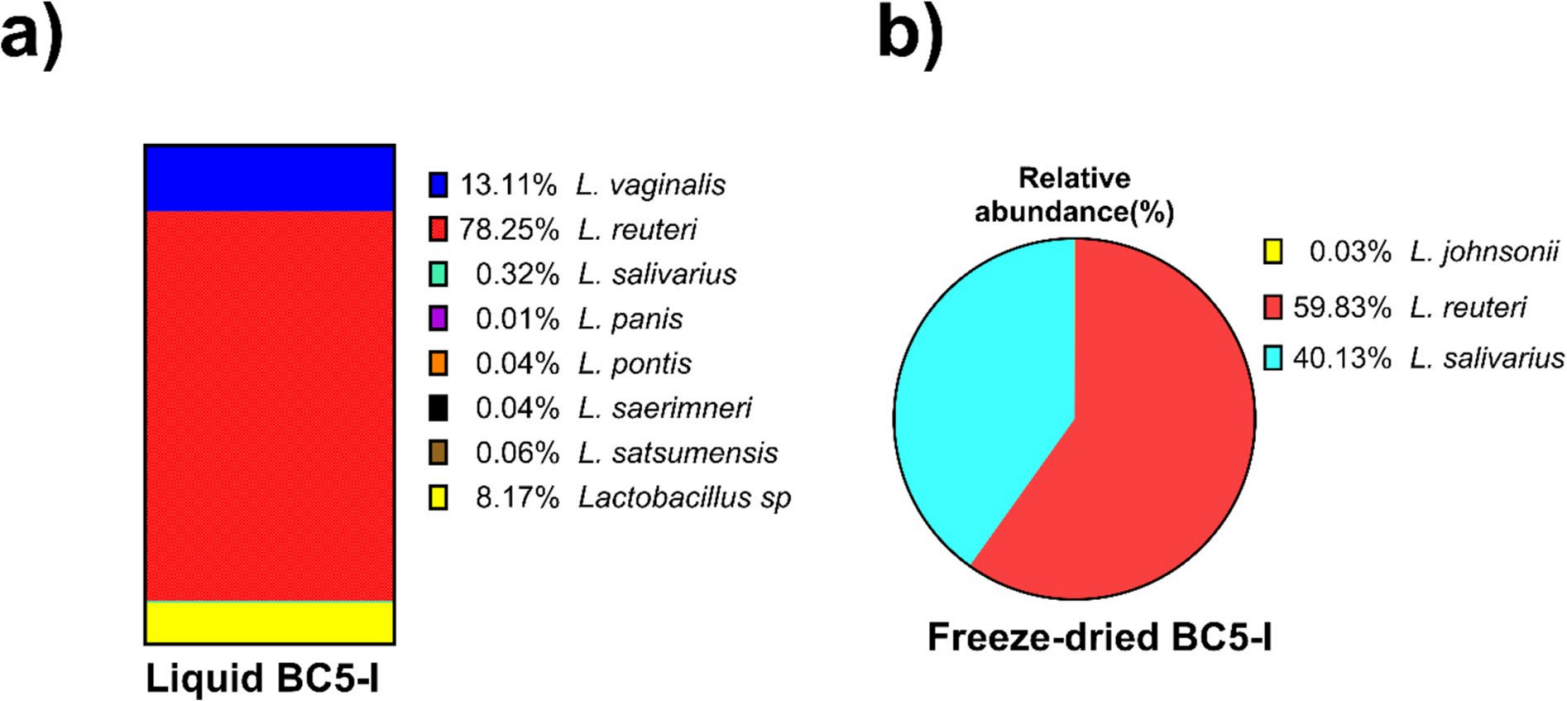
Relative abundance of *Lactobacillus* (%) at the species level in liquid (**a**) and freeze-dried (**b**) forms of BC5-I

After freeze-drying, a PCR analysis was conducted to identify specific *Lactobacillus* strains at the species level in BC5-I and BC7-I (Fig. 5). Both BC powders revealed the presence of *L. acidophilus*, *L. casei* (Fig. 5a), and *L. helveticus* (Fig. 5b). Nonetheless, *L. rhamnosus* was only present in BC7-I, while *L. cremoris* was only present in BC5-I. To corroborate these results, since freeze-dried BC5-I powders had the highest storage stabilities, 16S rRNA of both liquid and powders BC5-I forms was sequenced, and their relative abundance of species was analyzed (Fig. 6a, b). Interestingly, only the *Lactobacillus* genus was identified in both forms of BC5-I, resulting in three major *Lactobacillus* species in liquid form, including *L. reuteri* (78.25%), *Lactobacillus* sp. (8.17%), and *L. vaginalis* (13.11%). Conversely, freeze-drying changes the proportion of *Lactobacillus* species, with *L. reuteri* retaining the highest relative abundance (59.83%), followed by *Ligilactobacillus salivarius* (40.13%), and *L. johnsonii* (0.03%).

## Discussion

In this study, we characterized BC isolated from the GIT of 1-year-old clinically healthy wild-type chickens (*Gallus gallus*) to identify potential probiotics. Several tests were conducted to select BC according to the criteria needed for them to be included in the GRAS list, such as determining the microbial communities by 16S rRNA sequencing, antibiogram, resistance to GIT pH, temperature, simulated gastric juice, and mucus adhesion.

This work revealed 345 ASVs in the eighth chicken ileum and cecum samples BC (Tables S7 and S8). In agreement with previous findings, Proteobacteria and Firmicutes were the major phyla present. Interestingly, Fusobacteria were also present at low relative abundance (7%), especially in samples isolated from the cecum and exclusively in BC2-I from the ileum, which was unexpected since this phylum of Gram( −) bacteria has been previously associated with multifactorial diseases [34] and it is not commonly found in chicken gut microbiota. We hypothesize that its presence is associated with an opportunist infection in the farm [1]. Kollarcikova et al. [34] reported that Fusobacteria identified in the cecum of commercial hens (up to 20%) is an opportunistic pathogen associated with decreased egg production or the consequence of other causes related to immune-compromised individuals. In another study, Pandit et al. [35] demonstrated the presence of Fusobacterium (up to 43%) in commercial broilers. The authors concluded that it depends on the chicken breeds and lines and the geolocalization, indicating one or more environment-specific variables and offering opportunities for targeted genetic improvement by selective breeding [35].

Nevertheless, our experimental animals appeared clinically healthy and highlighted that the isolated consortia selected as probiotic candidates (BC5-I and BC7-I) do not contain Fusobacterium (Fig. 1a, Table S6). In contrast, at the order level, several studies reported the presence of Enterobacterales, Clostridiales, and Lactobacillales as common bacterial populations in chicken GIT [11, 15], which is consistent with our results. In addition, we found no alpha and beta diversity differences among BC isolated from the ileum and cecum of chickens (data not shown).

On the other hand, antibiograms are one of the most critical assays for evaluating AMR. Consequently, BC5-I and BC7-I samples isolated from the ileum were selected for this probiotic test, and the remaining samples were discarded. Additionally, both BC were grown through serial cultivation in selective medium MRS, which is a well-known method for isolating lactic acid bacteria while preventing the growth of Enterobacterales. Our colony morphology results corroborated that these BC did not contain enterobacteria such as *Salmonella*. The components of the MRS medium inhibited the growth of Gram( −) bacteria such as *Salmonella* and other pathogenic strains. These findings suggest that the DFM could be considered safer.

Another safety consideration in DFM studies is that a potential probiotic strain does not contain transferable antibiotic-resistance genes [3, 5]. Both BC5-I and BC7-I were sensible to the main Gram( +) and Gram( −) antibiotics tested. However, BC5-I and BC7-I revealed the presence in plasmid DNA of resistance genes to ampicillin (AM), vancomycin (VA), and aminoglycosides (AMG), which were previously reported in other probiotic strains [11, 36]. For example, Ma et al. [11] reported that the *L. reuteri* strain YPG14 isolated from the chicken GIT as potential probiotics was sensible to VA.

Mancabelli et al. [36] also demonstrated a relatively high abundance of AMR genes using a metagenomic approach in the resistome of chickens fed with antimicrobial supplements. These authors noted that VA and AMG showed significant incidences, suggesting that since these antibiotics have been widely used, the chicken microbiota incorporated these resistance genes. However, in our current work, except for VA, all tested antibiotics were effective. These results indicate that BC5-I and BC7-I have good safety characteristics and do not contain other transferable AMR genes, nor Fusobacteria and Enterobacteria pathogenic strains. Consistent with this, in preliminary studies in Bovans White chickens, these probiotics have exhibited no adverse health effects 2 months after their administration (data not shown).

Another crucial property of bacterial consortia, such as DFM, is their ability to survive the pH changes in the stomach and intestine and to colonize them [2, 9, 13]. The capability to tolerate acid and bile is considered a good indicator of the viability of the GIT. In addition, the probiotics should be tolerant to body temperatures, an essential parameter for their production and storage. These characteristics are often assessed in the preliminary examination of potential probiotic strains [9].

In our experiment, low pH (2.0 and 3.0) affected the growth of BC5-I and BC7-I (4–42%) at 24 h, although at pH > 4.0, satisfactory survival rates (96–102%) were observed. The survival of different strains depended on the exposure time and the pH values. In this sense, isolated probiotic strains from chicken GIT have shown controversial results [37–39]. For example, Kizerwetter-Swida et al. [37] reported a high tolerance (94–97%) of four Lactobacilli strains incubated at pH 2.0 for 4 h. Conversely, Hashemi et al. [38] described significantly lower viability of Lactobacilli after 2 h of incubation at the latter pH. Moreover, Aziz et al. [39] reported that *L. reuteri* and *L. johnsonii* strains were resistant to pHs 2.0 and 4.0, showing nearly 33% survival, which agrees with our results. Furthermore, both BCs in this work showed temperature tolerance, especially for BC5-I, which agrees with the literature [3, 24, 39].

The antimicrobial activity has been proposed to be essential for the ability of bacterial consortia to exert probiotic properties on their hosts [2, 8, 9]. BC5-I and BC7-I inhibited the growth of *Salmonella typhimurium* ATCC 19028 and *Escherichia coli* ATCC 11229, suggesting that these DFM could protect the chicken GIT against pathogenic bacteria and diseases that cause significant economic losses to the poultry industry. Our results converge with several studies that have demonstrated that DFM, especially Lactobacilli, can inhibit the growth of pathogenic strains such as *Salmonella enteritidis* [37], *S. pullorum* [11], *S. typhimurium* 14028 s [40], *Citrobacter*, *Listeria* [39], and *E. coli* [37]. Nonetheless, further research is still needed to elucidate the potential mechanisms of action underlying the antagonistic effects of our probiotics against pathogens and whether these effects are attributed to pH modification or the production of bacteriocins.

In our in vitro bile salt tolerance assay, BC5-I and BC7-I strains showed survival rates of 27% and 25%. These results align with previous studies indicating variability in *Lactobacillus* responses to bile salts [24, 41, 42]. For instance, some studies report that 10 out of 12 strains tested under 0.3% bile salt conditions showed delayed growth, while two strains were unaffected [41]. Furthermore, in another study, the bile salt tolerance of 184 *Lactobacillus* strains was determined resulting that 12% of *Lactobacillus* strains demonstrated high growth capacity in bile salt conditions, while 38% did not grow after 24 h, highlighting the heterogeneity in bile salt tolerance across strains. Bile salts act as weak acids with detergent-like properties, which can damage bacterial membranes, thereby limiting the growth of susceptible bacteria [42]. Our strains demonstrated moderate bile salt tolerance, suggesting their potential as probiotics capable of withstanding gastrointestinal stress. However, this in vitro data is supported by promising results from in vivo trials conducted in Bovans White chickens, where DFM supplementation at 10^8^ and 10^9^ CFU/mL improved intestinal health and increased body weight, with a low mortality rate of 1.2% (data not shown). These findings indicate the resilience of BC5-I and BC7-I and their potential efficacy as DFM products.

The simulated gastric digestion assay was performed as an indicator of the survival capacity of probiotics. This test showed that BC5-I and BC7-I could withstand the challenging conditions with a pH of 2.5 and exposure to pepsin for 2 h which mimic in vivo conditions. BC5-I displayed remarkable resilience, with only a slight decrease in viability. Our probiotics showed high resistance to SGD, similar to previous results obtained by other authors. In addition, Pokorná et al. [43] reported that different lactobacilli isolates from poultry have distinct resistance throughout the SGD time. These authors showed that after 30 min of the treatment, some Lactobacilli strains (e.g., *L*. *agilis*, *L. kitasatonis*, *L. plantarum*, among others) lost their viability, whereas *L. reuteri*, *L. acidophilus* (275), and *L. vaginalis* (683) showed high levels of viability through time [43]. Also, Ma et al. [11] noted that *L. reuteri* strains YPG14 and YPG16 isolated from chicken intestine were resistant to the SGD conditions. Our results indicate that BC5-I can be classified as a DFM candidate because it exerts the best tolerance for SGD conditions.

BC5-I was subjected to an in vitro chicken intestinal mucus adhesion test, showing an excellent capacity to adhere, which was 2.9 times higher than host mucosal bacteria. These findings are similar to other studies with lactobacilli strains [3, 11, 24]. The mucus produced in the intestinal epithelium plays a critical role in preventing pathogen entry across the epithelial barrier and serving as the primary site for microbiota adhesion and colonization [3]. The adhesion capacity of BC5-I would be helpful for its intestinal colonization and performance-related functional properties in experimental animals. This DFM was tested on the intestinal health of clinically healthy Bovans White birds at 56 days of age (data not shown). The findings have been promising, as no adverse effects from DFM supplementation have been observed regarding diarrhea and enteric diseases. Likewise, no cytotoxicity was observed in the histological analysis using hematoxylin and eosin staining of the small intestine, showing an improvement in the height and width of the intestinal villi. This can contribute to corroborating the safety of the DFM and the adhesion to chicken mucus and its possible beneficial effects; however, conclusive results that confirm it have yet to be obtained.

Regarding storage stability, BC5-I and BC7-I were spray- and freeze-dried, and their viability was evaluated during 30 days in storage. Although there was a decrease in viability at the beginning of storage with both drying methods, we demonstrated that freeze-drying was the best method for survival efficiency. Moreover, it prolonged the shelf-life of these powders compared to their counterparts in liquid form during the 30 days of storage. Under our experimental conditions, powders for BC5-I were satisfactorily obtained by both methods, especially those from freeze-drying, which exerted the best storage stabilities with the lowest rate of cell death (*K* = 0.004 days^−1^) and the highest half-life (*t*_1/2_ = 173.3 days). We associate a heat stress and dehydration effect on the spray-drying method as the principal cause of inactivation and loss of viability of BC5-I and poor production of BC7-I [17]. Despite this, the viability of BC5-I throughout the storage time remained constant but lower than in freeze-dried powders. This could be due to freezing the liquid probiotic with a CO_2_ ice bath before drying, which has been related to protecting the cell viability, leading to the formation of small crystals, thus avoiding extensive cellular damage and improving their viability [17]. Another reason for these results could be attributed to the moisture content and the water activity (aw) in the dry BC products. Both BC obtained by freeze-drying showed lower moisture content compared to those obtained by spray-drying. Several factors can compromise the probiotic viability during storage, including temperature, relative humidity, light exposure, and powder moisture [17, 20]. Low moisture is critical for maintaining long-term stability in probiotic products because high moisture could activate metabolic processes that are caused by cellular stress and oxidation of membranes, leading to reduced probiotic viability [17]. In this sense, freeze-dried probiotic products typically achieve lower moisture and aw than spray-dried, making them suitable for large-scale productions.

Other studies of DFM strains reported that freeze-drying is the best method to obtain dried products [17, 20, 44]. Moayyedi et al. [44] compared the effects of spray- and freeze-drying in *Lactobacillus rhamnosus* ATCC 7469 and described that freeze-drying was better for their survival even when exposed to SGD conditions. Ren et al. [20] assessed the production and storage of freeze-drying probiotic lactobacilli strains isolated from broilers using different protective agents (skim milk, sucrose, and trehalose). These authors found that the protectants confer higher storage survival rates and prolonged lactobacilli viability, effects that are strain-dependent.

In a previous study in our laboratory [18], we demonstrated that freeze-drying using skim milk as a cryoprotection agent provides the highest long-term storage protection of BC isolated from *Bos taurus*. The use of skim milk is known to be an excellent additional protection strategy during freeze-drying to preserving probiotics [17]. Skim milk stabilizes bacterial cell membranes and enables easier rehydration by creating a high-surface porous structure [20]. However, it is essential to acknowledge that our current study has limitations. There is still a need for a more in-depth investigation into the potential impacts related to the utilization of GA:WPC80 or skim milk as carrier agents, including a thorough exploration of its protective mechanisms, such as vitrification, and optimizing its role as a matrix. Additionally, other agents should be considered that might enhance the viability of the bacteria under examination [17].

Finally, the endpoint PCR confirmed the presence of various Lactobacilli strains in freeze-dried BC5-I (*L. acidophilus*, *L. helveticus*, *L. casei*, and *L. cremoris*), representing 73% of the total bacteria measured by qPCR. Furthermore, through 16S rRNA sequencing analysis, we identified that the freeze-dried BC5-I contains only three Lactobacilli strains, including *L. reuteri*, *L. salivarius*, and *L. johnsonii.* As previously stated, other studies have isolated these lactobacilli from the chicken intestine [11, 15, 20]. This reveals that the early microbiota communities in BC5-I changed and were selected using an MRS medium, which partly accounts for the alterations observed in the freeze-dried BC5-I. The MRS broth is a well-established medium for cultivating and selecting lactobacillus strains [20, 24].

The discrepancy between the results of endpoint PCR and Illumina sequencing for the analysis of BC5-I composition may be explained by fundamental differences in sensitivity, specificity, and range of both techniques. Endpoint PCR is a qualitative technique used to detect the presence or absence of specific DNA sequences. On the other hand, 16S rRNA sequencing provides more detailed information about the bacterial community present. In the case of BC5-I, it constitutes a more reliable and representative approach to understand the microbial diversity present. These results were well corroborated by the earlier colony morphology descriptions. Also, these results corroborated the efficacy of MRS medium to isolate and select a lactobacilli-based DFM free of *Salmonella* and other pathogenic strains. Therefore, these results reveal that both liquid and freeze-dried forms of BC5-I consist of *Lactobacilli* strains and that the dry product has desirable characteristics for its long-term shelf-life.

## Conclusions

The chicken gastrointestinal tract is an important source of bacterial communities with potential for the development of probiotics. BC5-I and BC7-I isolated from chicken ileum exhibited the best characteristics as candidates for DFM. They showed high antibiotic sensitivity, resistance to various GIT conditions such as pH and temperature, and antagonistic effects against pathogens. High resistance to the conditions of simulated gastric digestion and adherence to mucin capacities confirm that BC5-I displays good DFM properties. Likewise, their lyophilization resulted in a dry product with satisfactory survival efficiency and shelf-life stability. Therefore, BC5-I is a lactobacilli-based strain that exhibits favorable safety for poultry administration. It offers potential benefits as it could enhance growth performance, animal health, and superior quality of chicken meat and eggs. Nevertheless, further research into the specific protective mechanism of BC5-I is necessary to elucidate its effects on the host and promote its applications.

## Supplementary Information

Below is the link to the electronic supplementary material.

Supplementary file1 (PDF 388 KB)

Supplementary file2 (XLSX 29 KB)

Supplementary file3 (XLSX 23 KB)

## Data Availability

"Raw data reported in this study can be found in BioProject PRJNA1033643 of the Sequence Read Archive (SRA) database of the NCBI. Additional data that support the findings of this study are available as supplementary material."
