# Supplementary material for: Isolation, Potential Beneficial Properties, and Assessment of Storage Stability of Direct-Fed Microbial Consortia from Wild-Type Chicken Intestine
Source: Probiotics Antimicrob Proteins. 2024 Oct 25;17(6):4508–24. doi: 10.1007/s12602-024-10387-0 (PMC12634797; doi:10.1007/s12602-024-10387-0)
Supplement: Supplementary file 1 — Supplementary file1 (PDF 388 KB) [file 12602_2024_10387_MOESM1_ESM.pdf]

Supplementary Information

Isolation, potential beneficial properties, and storage stability assessment of direct-fed microbial consortium from wild-type chickens' (*Gallus gallus*) intestines

Probiotics and Antimicrobial Proteins

Haiku D. J. Gómez-Velázquez [1], Pamela Peña-Medellín [2], Carlos O. Guzmán-Hernández [2], Laura González-Dávalos [1], Alfredo Varela-Echavarría [3], Armando Shimada [1] and Ofelia Mora[1]*

[1] Laboratorio de Rumiología y Metabolismo Nutricional (RuMeN), Facultad de Estudios Superiores-Cuautitlán (FESC)-UNAM, Querétaro 76231, Qro., Mexico
[2] Posgrado en Ciencias de la Producción y la Salud Animal, Universidad Nacional Autónoma de México (UNAM), Mexico
[3] Instituto de Neurobiología, Universidad Nacional Autónoma de México, Querétaro, 76231, Qro., Mexico
*Correspondence: ofemora66@unam.mx

Table S1 Primer sequence for specific antibiotic resistance genes

Antibiotic	Primer name	Sequence (5'- 3')	Annealing temp (°C)
**Ampicillin (AM)**	502 F	AATGAAGCCATACCAAACGAC	54.7
	632 R	CGGATAAAGTTGCAGGACCA	
**Chloramphenicol (CL)**	387 F	CGTTTATGCCAACCGTCCTG	57.3
	525 R	GGCTATACTGGCGATGCT	
**Erythromycin (E)**	300 F	GATATTTGGAAGTATACCCT	47.7
	521 R	GTGTCTTAATAAAACTTACCCG	
**Vancomycin A (VA-A)**	382 F	GGGAAAACGACAATTGC	52
	615 R	GTACAATGTGGCCGTTA	
**Vancomycin B (VA-B)**	420 F	ATGGGAAGCCGATAGTC	48
	612 R	GATTTCGTTCCTCGACC	
**Aminoglycosides (AMG)**	194 F	TAATGCCTACTCAGACCA	58.7
	409 R	AAGTATCTCGCTAATTCACC	
**Tetracycline M (TE-M)**	408 F	GTGGACAAAGGTACAACGAG	64
	R	CGGTAAAGTTCGTCACACAC	
**Tetracycline W (TE-W)**	F	GAGAGCCTGCTATATGCCAGC	64
	R	GGGCGTATCCACAATGTTAAC	

Table S2 Primer sequence for *Lactobacillus* at the species level

Target	Primer name	Sequence (5'- 3')	Annealing temp (°C)	Amplicon size (bp)
Lactobacillus acidophilus	496 F<br>692 R	CAACCAGAAAGTCACGGCTA<br>ATCTACGCATTCCACCGCTA	57.0	302
Lactobacillus casei	606 F<br>781 R	AGTCTGATGTGAAAGCCCTC<br>ATGGGTAGCGAACAGGATTAG	56.6	175
Lactobacillus helveticos	611 F<br>786 R	ATGCTGATGTGAAAGCCCTC<br>CTAATCCTGTTCGCTACCCAT	56.2	175
Lactobacillus rhamnosus	995 F<br>1298 R	CTTCCGGTACGAACAACTCT<br>AGTCCGGTGTTTGGTTTAGC	57.0	176
Lactobacillus cremoris	1091 F<br>1278 R	CCCCTATTGTTAGTTGCCAT<br>TTGCAGCCTACAATCCGAAC	54.8	207

Table S3 Specific primer design for proportion bacteria identification by qPCR

Target	Primer name	Sequence (5'- 3')	Annealin g temp (°C)	Reference
**Total Bacteria (universal primers)**	bact1369F<br>Prok1492R	CGGTGAATACGTTCYCGG<br>GGWTACCTTGTTACGACTT	56	Suzuki et al. [1]
**Total *Lactobacillus***	F<br>R	AGCAGTAGGGAATCTTCCA<br>CACCGCTACACATGGAG	56	Rinttilä et al. [2]
**Total *Enterobacteria***	F<br>R	ATGTTACAACCAAAGCGTAC A<br>TTACCYTGACGCTTAACTGC	56	Takahashi et al. [3]

Table S4 Summary of 16S rRNA gene sequencing reads for chicken gastrointestinal tract bacteria across samples

Samples	Sample type	Input Raw reads	Filtered	Denoised Forward	Denoised Reverse	Merged	Non chimeras	Merged (%)
**BC10C**	**Cecum**	28463	11053	10835	10895	10122	7690	76.0
**BC1C**	**Cecum**	37729	14083	13936	14001	13510	12029	89.0
**BC2I**	**Ileum**	24180	9838	9451	9702	8963	6962	77.7
**BC3C**	**Cecum**	17216	6749	6535	6559	6187	4940	79.8
**BC4I**	**Ileum**	29339	13621	13454	13516	13083	7520	57.5
**BC5I**	**Ileum**	29489	11446	11298	11317	10803	8754	81.0
**BC6C**	**Cecum**	24108	9390	9341	9382	9173	8229	89.7
**BC7I**	**Ileum**	25633	10237	10178	10149	9974	8573	86.0
**Total**		216157	86417	85028	85521	81815	64697	79.1

Table S5 Bacterial relative abundance (%) at phyla level (top 10) of ileum and cecum chicken intestine

Phyla	Samples							
	Cecum				Ileum			
	BC10C	BC1C	BC3C	BC6C	BC2I	BC4I	BC5I	BC7I
**Firmicutes**	44.486346	17.1335938	16.82186	13.52534	38.063775	100	41.29541	8.596757
**Fusobacteriota**	2.873862	0.9643362	7.08502	0	3.389831	0	0	0
**Proteobacteria**	52.639792	81.90207	76.09312	86.47466	58.546395	0	58.70459	91.403243

Table S6 Bacterial relative abundance (%) at the order level (top 10) of ileum and cecum chicken intestine

Order	Samples							
	Cecum				Ileum			
	BC10C	BC1C	BC3C	BC6C	BC2I	BC4I	BC5I	BC7I
**Lactobacillales**	2.87	13.45	11.88	13.28	15.38	10.17	30.10	5.027
**Clostridiales**	39.57	0	3.64	0.24	9.55	85.80	5.837	0.875
**Lachnospirales**	0	0	0	0	0	0.29	0	0
**Oscillospirales**	1.743	0.91	1.30	0	2.03	0	0	0
**Peptostreptococcales-Tissierellales**	0.30	2.77	0	0	11.10	3.74	5.36	2.70
**Fusobacteriales**	2.87	0.96	7.09	0	3.39	0	0	0
**Enterobacterales**	52.64	81.90	76.09	86.48	58.55	0	58.71	91.40

References

1. Suzuki MT, Taylor LT, DeLong EF (2000) Quantitative analysis of small-subunit rRNA genes in mixed microbial populations via 5'-nuclease assays. Appl Environ Microbiol 66:4605–4614. https://doi.org/10.1128/AEM.66.11.4605-4614.2000

2. Rinttilä T, Kassinen A, Malinen E, et al (2004) Development of an extensive set of 16S rDNA-targeted primers for quantification of pathogenic and indigenous bacteria in faecal samples by real-time PCR. J Appl Microbiol 97:1166–1177. https://doi.org/10.1111/j.1365-2672.2004.02409.x

3. Takahashi H, Saito R, Miya S, et al (2017) Development of quantitative real-time PCR for detection and enumeration of Enterobacteriaceae. Int J Food Microbiol 246:92–97. https://doi.org/10.1016/j.ijfoodmicro.2016.12.015
